# Improved Immune Responses in Young and Aged Mice with Adjuvanted Vaccines against H1N1 Influenza Infection

**DOI:** 10.3389/fimmu.2018.00295

**Published:** 2018-02-19

**Authors:** Susan L. Baldwin, Fan-Chi Hsu, Neal Van Hoeven, Emily Gage, Brian Granger, Jeffrey A. Guderian, Sasha E. Larsen, Erica C. Lorenzo, Laura Haynes, Steven G. Reed, Rhea N. Coler

**Affiliations:** ^1^Infectious Disease Research Institute, Seattle, WA, United States; ^2^Center on Aging, Department of Immunology, University of Connecticut School of Medicine, Farmington, CT, United States; ^3^Department of Global Health, University of Washington, Seattle, WA, United States; ^4^PAI Life Sciences, Seattle, WA, United States

**Keywords:** influenza, vaccine, adjuvant, elderly, T helper 1, H1N1

## Abstract

Elderly people are at high risk for influenza-related morbidity and mortality due to progressive immunosenescence. While toll-like receptor (TLR) agonist containing adjuvants, and other adjuvants, have been shown to enhance influenza vaccine-induced protective responses, the mechanisms underlying how these adjuvanted vaccines could benefit the elderly remain elusive. Here, we show that a split H1N1 influenza vaccine (sH1N1) combined with a TLR4 agonist, glucopyranosyl lipid adjuvant formulated in a stable oil-in-water emulsion (GLA-SE), boosts IgG2c:IgG1 ratios, enhances hemagglutination inhibition (HAI) titers, and increases protection in aged mice. We find that all adjuvanted sH1N1 vaccines tested were able to protect both young and aged mice from lethal A/H1N1/California/4/2009 virus challenge after two immunizations compared to vaccine alone. We show that GLA-SE combined with sH1N1, however, also provides enhanced protection from morbidity in aged mice given one immunization (based on change in weight percentage). While the GLA-SE-adjuvanted sH1N1 vaccine promotes the generation of cytokine-producing T helper 1 cells, germinal center B cells, and long-lived bone marrow plasma cells in young mice, these responses were muted in aged mice. Differential *in vitro* responses, dependent on age, were also observed from mouse-derived bone marrow-derived dendritic cells and lung homogenates following stimulation with adjuvants, including GLA-SE. Besides enhanced HAI titers, additional protective factors elicited with sH1N1 + GLA-SE in young mice were observed, including (a) rapid reduction of viral titers in the lung, (b) prevention of excessive lung inflammation, and (c) homeostatic maintenance of alveolar macrophages (AMs) following H1N1 infection. Collectively, our results provide insight into mechanisms of adjuvant-mediated immune protection in the young and elderly.

## Introduction

Influenza and influenza-related complications are leading causes of death in elderly populations. Older people exhibit increased morbidity and mortality in response to influenza infection due to uncontrolled respiratory inflammation, severe pneumonia, or multi-organ inflammation and failure (1). Although the annual influenza vaccine coverage rate has increased, the CDC estimates that individuals older than 65 years of age who have received influenza vaccines in consecutive years are still at high risk of influenza infection (2).

The process of immunological aging, also called immunosenescence, is associated with a progressive loss of functional physiological integrity including collectively increasing DNA damage and genome instability, stem cell exhaustion, cellular senescence, and altered intercellular communication, among other processes [reviewed in Ref. (3)]. Immunosenescence causes the attenuation of both innate and adaptive immune systems including reduced levels and function of TLRs in macrophages and plasmacytoid dendritic cells (4, 5), decreased output of naïve B cell numbers (6), severe reduction of thymopoiesis (7), and insufficient immune synapse generation (8). Furthermore, aged individuals also demonstrate a significant reduction in the quantity, but not quality of antigen-specific antibody responses, and this reduction in antibody quantity is concurrent with a decrease in antigen-specific plasmablasts (9). Indeed, defective antigen presentation and reduced T cell and B cell repertoires in aged individuals results in poor cellular, humoral, and vaccine-induced responses.

As vaccination is the most effective method to prevent infection, ways to improve influenza vaccines for use in the aging human population are continually being explored. These strategies include adjuvant use, intradermal delivery, increased dosage, and altering antigen selection that typically make up seasonal vaccines [reviewed in Ref. (10)]. The first seasonal influenza vaccine containing an adjuvant (MF59), also known as Fluad™, was approved by the FDA in 2015 for use in people over the age of 65 years. Fluzone^®^ High-Dose vaccine (an inactivated, split influenza virus vaccine containing 60 µg of HA for each component of the vaccine, rather than 15 µg) is also approved for use in people ≥65 years of age. Despite advancements in alternative vaccine options for the elderly population, they are still disproportionally affected and continue to exhibit severe morbidity and mortality each year from seasonal influenza infections. Thus, a need still remains for better strategies, including additional adjuvant options, for the influenza vaccine to overcome the challenges of immunosenescence in the elderly.

The disparity of morbidity and mortality burden in elderly individuals is partly due to the incomplete understanding of how adjuvanted vaccines may circumvent immunosenescence in elderly people. Mechanistic details regarding how different adjuvants work in the elderly may reveal ways in which adjuvant formulations can be tailored and adapted for optimal responses that provide enhanced protection for this susceptible population. Administration of adjuvants, including squalene-based oil-in-water emulsions, and those including a TLR4 agonist, have been leveraged in vaccines in order to enhance immune responses in multiple studies (11–18). These adjuvants include MF59 (as described above), and AS04 (comprised of monophosphoryl lipid A and Alum) included in FDA approved vaccines against hepatitis B virus and human papillomavirus (11). In preclinical studies, we have shown that glucopyranosyl lipid A (a synthetic TLR4 agonist) formulated in a stable oil-in-water emulsion (GLA-SE) expands immune responses to Fluzone^®^ (12). GLA has also been included in several completed and ongoing human clinical studies for vaccines against *Leishmania* (NCT01751048), *Mycobacterium tuberculosis* (NCT02508376, NCT0246516, NCT01599897), HIV (NCT01966900, NCT01922284), Schistosomiasis (NCT03041766), malaria (NCT02647489, NCT01540474), and avian influenza (NCT01991561, NCT01147068). In humans, GLA-SE combined with rH5 was considered safe and improved antibody titers compared to the recombinant protein alone (15). Furthermore, no defect has been identified in humans following stimulation with GLA-SE on antigen-presenting cells (APCs) from aged compared to young APCs (16) and enhances T cell responses from older adults following stimulation of peripheral blood mononuclear cells (PBMCs) with live influenza virus (19). Therefore, our approach in this study was to employ the use of different adjuvants combined with an influenza vaccine to overcome the challenge of immunosenescence in aged animals.

In this investigation, one of our main findings reveal that aged mice have dampened cytokine responses to *in vitro* stimulation of DCs and lung homogenates with a T helper 1 (Th1) cytokine-inducing agonist. In addition, we show that aged mice require two immunizations with adjuvanted sH1N1 vaccines for robust *in vivo* protection against influenza whereas young mice are protected after a single immunization. Finally, we demonstrate that immunization in young mice with sH1N1 + GLA-SE results in enhanced alveolar macrophage (AM) homeostasis within the lung, increased TLR7 expression within the AMs, and faster clearance of virus after H1N1 challenge compared to mice immunized with sH1N1 + SE or vaccine alone. These results emphasize the age-related differences following immunization, the ability to improve responses to influenza infection in the elderly with two immunizations, and a mechanism of enhanced vaccine protection against influenza with a TLR4 agonist adjuvant.

## Materials and Methods

### Mouse Model

Young female C57BL/6 or CB6F1 mice were purchased from Charles River Laboratories (Wilmington, MA, USA) or the Jackson Laboratories (Bar Harbor, ME, USA) and were housed and maintained under specific pathogen free conditions at the Infectious Disease Research Institute. Experiments that included female C57BL/6 or CB6F1 mice aged 18–21 months were acquired with special request from the National Institute on Aging, from the aged rodent colony (National Institute of Health, Bethesda, MD, USA). Mice were housed in a biosafety level 2 (BSL2) environment for the entirety of these studies (including H1N1 challenge studies), and all procedures were performed in accordance with the regulations and guidelines of the IDRI animal care and use committee.

### Adjuvants and Immunization

Vaccines were formulated in saline, SE (2% final v/v oil concentration), an MF59-like adjuvant (2.5% final v/v oil concentration), or GLA-SE (5 µg of GLA in 2% SE). Mice were immunized intramuscularly (i.m.) one or two times 3 weeks apart. The split H1N1 influenza vaccine (sH1N1) (kindly provided by Novartis) and recombinant H1 (rH1) (A/California/4/2009; Protein Sciences Corp., Meriden, CT, USA) was used at 0.01 or 0.1 µg, respectively, for the immunizations. Serum was collected 3 weeks after prime or boost immunizations. Spleens and bone marrow were harvested for immunogenicity studies either 1 or 4 weeks following immunization(s) as described.

### Endpoint Antibody Titers

Sera were analyzed for H1-specific IgG1 and IgG2c endpoint antibody titers by antibody capture ELISA. Polysorp ELISA plates (Nunc-immuno polysorp 96 well plates, VWR) were coated with rH1 (A/H1N1/California/2009) (Protein Sciences Corp., Meriden, CT, USA) at a concentration of 1 µg/ml in 0.1 M bicarbonate coating buffer at 100 µl per well for 4 h at room temperature. Plates were then blocked with a 0.05% PBS-Tween solution plus 1% BSA and incubated overnight at 4°C, followed by five washes in 0.1% PBS-Tween and one PBS wash. Serially diluted mouse sera were added and plates incubated at room temperature, on a shaker, for 2 h. Plates were washed, dried, and secondary antibodies [IgG1-horseradish peroxidase (HRP) and IgG2c-HRP, Southern Biotechnologies, Birmingham, Al, USA] were added at a 1:2,000 dilution. Plates were incubated at room temperature for 1 h, washed, and eBioscience™ TMB solution (Thermo Fisher Scientific) was added to the plates. The enzymatic reaction was stopped with 50 µl per well of 1N H_2_SO_4_. Plates were then read on a VERSAmax microplate reader (Molecular Devices) at 450 nm with a reference filter set at 570 nm. Endpoint titers were determined as the last dilution to render a response of greater than 0.1 mean optical density using Prism software (GraphPad Software, La Jolla, CA, USA).

### Hemagglutination Inhibition (HAI) Antibody Responses

Hemagglutination inhibition assays were performed according to World Health Organization guidelines. Briefly, sera was treated with receptor destroying enzyme (from *Vibrio cholerae*, Denka-Seiken, Tokyo, Japan) overnight and heated at 56°C for 30 min to deactivate the enzyme. HAI antibodies were then tested against the vaccine strain (A/H1N1/California/4/2009) with 0.5% turkey red blood cells (Thermo Fisher Scientific). The HI titer was defined as the reciprocal of the highest dilution of sera, which completely inhibited the agglutination of the RBCs. All samples were run in duplicate, and pre-immune titers in all mice were ≤5.

### Weight and Survival Measurement

Ten mice/group were immunized i.m. either once or twice, 3 weeks apart. Three weeks after the prime or boost immunization, mice were infected *via* the intranasal route with 100LD_50_ A/H1N1/California/4/2009. Survival and clinical signs (weights) were assessed daily, over 14 consecutive days. Animals exhibiting significant weight loss or that were under duress (ruffled fur, weighted breathing, or hunched backs) were sacrificed. All procedures with A/H1N1/California/4/2009-infected mice were performed under BSL2 conditions per IACUC procedures and guidelines.

### *In Vitro* Stimulation of Bone Marrow Dendritic Cells (BMDCs) and Lung Homogenates

Bone marrow was harvested from the femurs of vaccinated mice, and single cell suspensions were prepared at a concentration of 2 × 10^5^ cells/ml in complete RPMI-1640 with 20 ng/ml recombinant mouse GM-CSF (rmGM-CSF; PeproTech). On day 3, additional rmGM-CSF was supplied. BMDCs were harvested on day 6 and were seeded at a concentration of 6.6 × 10^5^ cells per well. Cells were stimulated with a range of 2–20 µg/ml GLA-SE, 0.00008–0.8% SE, or 10 ng/ml Lipid A 506 (a TLR4 agonist as positive control, Peptide Institute Inc., Osaka, Japan) in the presence of 20 ng/ml rmGM-CSF. A sterile single cell suspension was prepared from young and aged mouse lung by passing tissue through a 100 µM cell strainer (Thermo Fisher Scientific). Erythrocytes were lysed using ACK lysis buffer (Gibco by Life Technologies), while remaining leukocytes were enumerated and plated in triplicate wells at a seeding density of 5 × 10^5^ cells per well. Lung homogenates were stimulated with the same conditions as BMDCs described above. Following an 18-h stimulation, cell supernatants from BMDCs or lung homogenates were collected, and cytokine levels in the supernatants were determined by using custom Luminex-based multiplex immunoassay kits (Procarta Cytokine Assay Kit: Affymetrix, Santa Clara, CA, USA). Cell supernatants were incubated with polystyrene beads coated with antibodies corresponding to the different cytokines including: TNF-α, IL-10, IL12p40, and IL-6 and developed according to the manufacturer’s instructions. Bead size and fluorescence were measured on a Luminex 200 and data were analyzed using the Masterplex QT software (Miraibio).

### Flow Cytometry

The antigen-specific T cell memory response generated by vaccination was determined by flow cytometry following incubation of splenocytes with 10 µg/ml rH1 (Protein Sciences Corp., Meriden, CT, USA). Cells were cultured at 1 × 10^6^ cells per well in a 96-well plate (Corning Incorporated, Corning, NY, USA) in RPMI-1640 supplemented with 10% heat-inactivated FCS and 50,000 U penicillin/streptomycin (Invitrogen) for 18–20 h in the presence of GolgiStop (BD Bioscience). Each sample was incubated with Fc receptor blocking (clone2.4G2) before incubation on ice for 30 min with the following antibodies: anti-CD4 (clone RM4-5), anti-CD8α (clone 53-6.7), and anti-CD44 (clone IM7). Expression of selected cytokines was determined by incubation with anti-IFN-γ (clone XMG1.2), anti-IL-2 (clone JES6-5H4), anti-CD154 (clone MRI), anti-TNF-α (clone MP6-XT22), and anti-IL-17A (clone TC11-18H10.1). For Tfh cell analysis, single-cell suspension of inguinal lymph nodes (20) were prepared and stained with following antibodies: anti-CD4 (clone RM4-5), anti-CXCR5 (clone L138D7), anti-PD-1 (clone 29F.1A12), anti-B220 (clone RA3-6B2), anti-CD44 (clone IM7), and anti-CD8 (clone 53-5.8), F4/80 (clone BM8), CD11b (clone M1/70) for dump gate. For germinal center (GC) B cell analysis, single-cell suspension of spleen or inguinal LNs were prepared and stained with following antibodies: anti-B220 (clone RA3-6B2), anti-GL7 (clone G7), anti-CD95 (clone SA367H8), anti-IgD (clone 11-26c.2a), anti-IgG1 (clone RMG1-1). For lung immune cell analysis, lungs were chopped and digested in the presence of 70 µg/ml Liberase™ (Roche), 40 µg/ml DNase I (Roche), 10 mM Aminoguanidine (Sigma-Aldrich), and 5 mM KN-62 (Sigma-Aldrich). The homogenates were incubated for 30 min in a 37°C water bath. Single-cell suspensions were prepared by dispersing the tissues through a 70 µm nylon tissue strainer (BD Falcon). Cells were washed with 1× PBS and then stained with a fixable viability dye (Tonbo Biosciences) before being stained with following antibodies: anti-CD11b (clone M1/70), anti-CD11c (clone N418), anti-CD45 (clone 30-F11), anti-NK1.1 (clone PK136), anti-Ly6G (clone 1A8), anti-TLR2 (clone T2.5), anti-TLR3 (clone 11F8), anti-TLR4 (clone SA15-21), and anti-TLR7 (clone A94B10). Antibodies were purchased from BD Biosciences, eBioscience, BioLegend, or Tonbo Biosciences. Samples were analyzed with BD Fortessa or LSRII. Doublets and dead cells were excluded before analysis, and all the data were analyzed with FlowJo software (version 9; FlowJo, LLC).

### Enumeration of Long-Lived Antibody-Secreting Plasma Cells (ASPCs)

A bone marrow ELISPOT was used to determine the induction of vaccine-specific long-lived ASPCs following immunization with sH1N1 vaccine with and without adjuvants. ELISPOTs were performed as previously described (21) with minor revisions. The developed plates were counted by an ELISPOT plate reader (C.T.L. Serie3A Analyzer, Cellular Technology Ltd., Cleveland, OH, USA), and the data were analyzed using ImmunoSpot^®^ software (Cellular Technology Ltd., Cleveland, OH, USA).

### Viral Load Measurement by Real-time Q-PCR

RNA from whole-lung samples was extracted with Trizol^®^ reagent following the manufacturer’s instructions (Thermo Fisher Scientific). The mixture of CHCl_3_ and Trizol was centrifuged at 11,500 *g* for 15 min at 4°C. After centrifugation, the aqueous layer was transferred and mixed with an equal volume of 70% RNase-free ethanol. The RNA extracts were purified with an RNA purification kit (Ambion^®^, Thermo Fisher Scientific). RNA was then reverse-transcribed with a SuperScript^®^ IV first-strand synthesis system (Invitrogen^®^, Thermo Fisher Scientific) into cDNA. Expression of H1 was measured using customized TaqMan probes (Applied Biosystem, Thermo Fisher Scientific) including forward primer: 5′-ATTGCCGGTTTCATTGAAGG-3′; reverse primer: 5′-ATGGCATTCTGTGTGCTCTT-3′; probe: 5′-(FAM) ATGAGCAGGGGTCAGGATATGCAGCCGACC (TAMRA)-3′ to detect A/H1N1/California/4/2009 viral load (22). TaqMan probe for GAPDH was used as the internal control (Applied Biosystem, Thermo Fisher Scientific). Samples were analyzed using a Bio-Rad CFX384 real-time PCR detection system (Bio-Rad), and relative gene expression was calculated *via* the 2^−ΔΔCT^ method.

### Statistical Analysis

Statistical analysis of antibody responses (endpoint antibody and HAI titers) and flow cytometry data was performed using one-way ANOVA with the Tukey multiple comparison test unless noted in the figure legend. Statistical analysis of cytokine production from BMDCs and lung homogenates represented in Figure 4 were performed using two-way ANOVA and Sidak’s posttest. A two-way ANOVA with the Tukey multiple comparison test was performed on data represented in Figure 5 (with the exception of Figure 5A; which used one-way ANOVA and the Tukey multiple comparison test). Statistical analysis of survival curves was performed using the Log-Rank/Mantel–Cox test. All statistical analyses were performed using GraphPad Prism version 7 for Windows (GraphPad Software, La Jolla, CA, USA). *p-*Values of <0.05 were considered significant.

## Results

### sH1N1 Vaccine Adjuvanted with GLA-SE Enhances IgG2c:IgG1 Ratios and High HAI Titers in Young CB6F1 Mice

We were particularly interested in determining whether adjuvants could help overcome the immunosenescence observed in elderly populations. The induction of antigen-specific IgG1 antibodies is dependent on Th2-biased immune responses, while class switching to IgG2c in mice is correlated with Th1-biased immune responses and has been well studied (20). We have previously reported that GLA-SE enhances antiviral protection through the induction of Th1-mediated immune responses (14) and, therefore, used this adjuvant formulation in this study. C57BL/6 mice were originally selected as the mouse strain for these studies; however, our data showed no evidence of HA (A/H1N1/California/4/2009) CD4 T cell epitopes in C57BL/6 mice, whereas CB6F1 mice have a confirmed CD4 T cell epitope (manuscript in preparation). These data provided the rationale for switching to the CB6F1 mouse strain for the remainder of the studies. As shown in Figure S1 in Supplementary Material, the recombinant rH1 vaccine combined with adjuvants MF59-like, SE, or GLA-SE in C57BL/6 mice induced both IgG1 and IgG2c responses post boost. An enhanced IgG2c:IgG1 bias was observed in C57BL/6 mice immunized with rH1 + GLA-SE (Figure S1B in Supplementary Material). While HAI titers in young C57BL/6 mice given adjuvanted rH1 were all >1:40 after two immunizations, much lower HAI titers were observed in aged C57BL/6 mice, where only one out of three of the aged animals in the MF59 and GLA-SE adjuvanted groups tested had post-vaccination HAI titers of >1:40 (Figure S1C in Supplementary Material).

**Figure 1:**
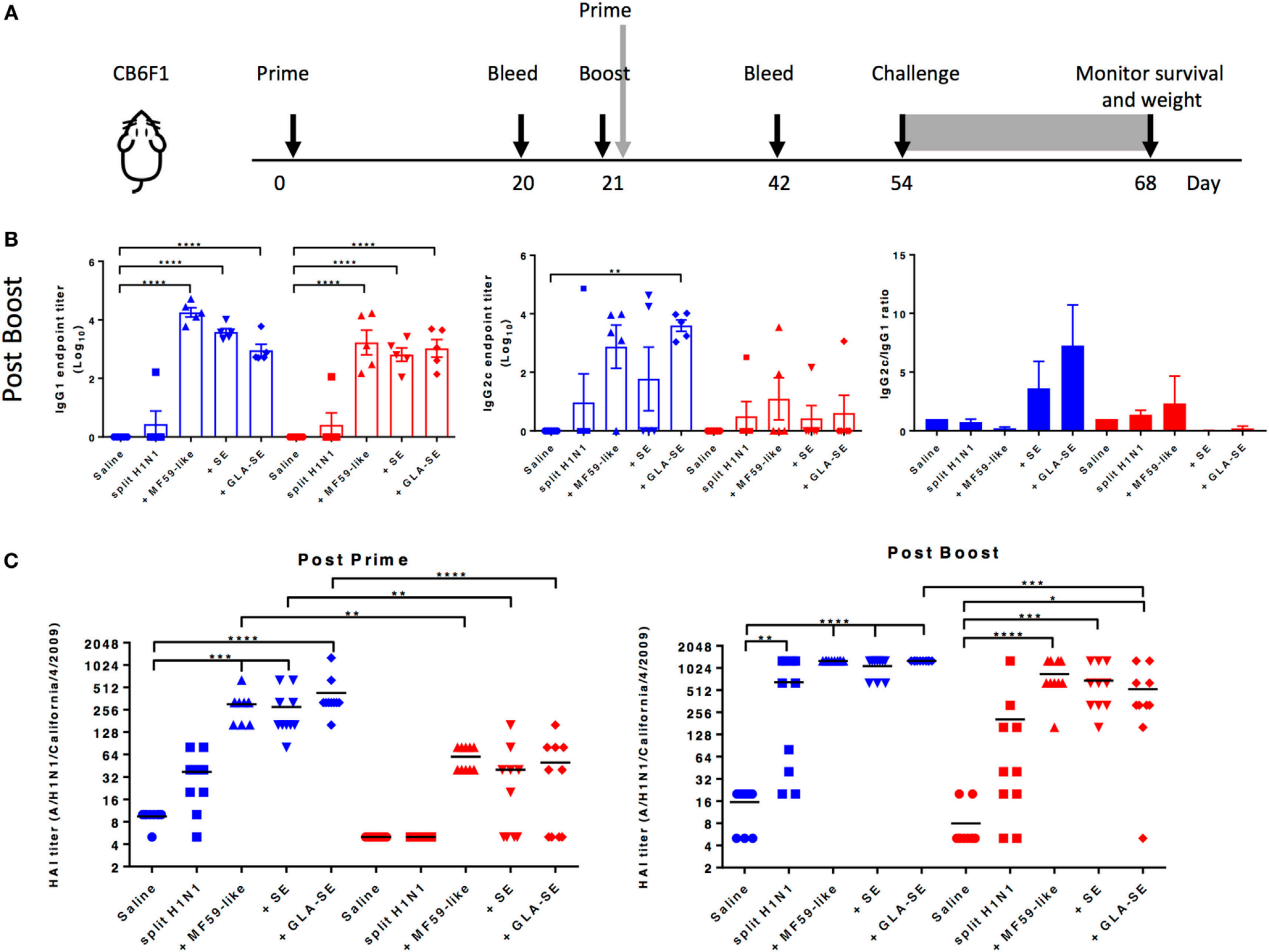
Adjuvanted sH1N1 vaccines induce antigen-specific IgG1 and IgG2c antibodies in young mice and hemagglutination inhibition (HAI) titers in young and aged CB6F1 mice. **(A)** Scheme of immunization procedure: CB6F1 mice were immunized i.m. either once or twice, 3 weeks apart, and antibody analysis was determined on sera collected 3 weeks following immunization. All mice were challenged with 100LD_50_ A/H1N1/California/4/2009 at day 54, and their physical condition was monitored over 14 days. Data for young mice were color-coded as blue; aged mice were color-coded as red for entire figure. **(B)** Sera from saline, sH1N1, sH1N1 + MF59-like, sH1N1 + SE, and sH1N1 + GLA-SE groups were analyzed for H1-specific IgG1 and IgG2c endpoint titers. Results are represented as the mean endpoint titer (log10) ± SEM. ** indicates *p-*value <0.01; *** indicates *p-*value <0.001; **** indicates *p-*value <0.0001. **(C)** Sera harvested from mice after a prime (day 20) or boost (day 42) immunization were analyzed for HAI titers. An HAI titer of five was assigned to responses below the assay detection limit. *p-*Values are denoted as follows: * indicates <0.05; ** indicates <0.01; *** indicates < 0.001; **** indicates < 0.0001.

We used the experimental design outlined in Figure 1A to assess the use of adjuvants in sH1N1 vaccine regimens and evaluate induced adaptive immune responses. Young (1 month old) or aged (18–21 months old) CB6F1 mice were immunized twice with the sH1N1 vaccine control (saline), or adjuvanted with MF59-like adjuvant, SE, or GLA-SE. Three weeks following the boost immunization, H1-specific IgG1 and IgG2c antibodies were evaluated (Figure 1B). We found that both young and aged mice receiving adjuvanted sH1N1 vaccines demonstrated measurable induction of IgG1 post boost over that of sH1N1 vaccine alone. However, the induction of IgG2c titers after two immunizations in aged mice were compromised even in adjuvanted groups, suggesting that the Th1-biased antibody response in aged mice, with the reduced dose of sH1N1 vaccine (shown to be effective in young mice), is not easily overcome by the action of adjuvants. Next, we tested whether adjuvanted sH1N1 vaccines could induce protective neutralizing antibodies in both young and aged mice. We observed that aged mice, which received MF59-like, SE, and GLA-SE adjuvanted vaccines could induce protective immune responses with two immunizations as compared to saline or sH1N1 vaccine alone, but not with one immunization (Figure 1C). These data suggest that adjuvants similar to MF59-like, SE, and GLA-SE could induce protective HAI titers in aged mice with a boost immunization; however, a boost with the TLR4 agonist adjuvant (GLA-SE) was unable to generate a Th1-biased environment capable of inducing IgG2c responses in aged mice with the sH1N1 vaccine.

### Enhanced Body Weights and Survival in Young and Aged CB6F1 Mice Following Two Immunizations with Adjuvanted sH1N1 Vaccines

**Figure 2:**
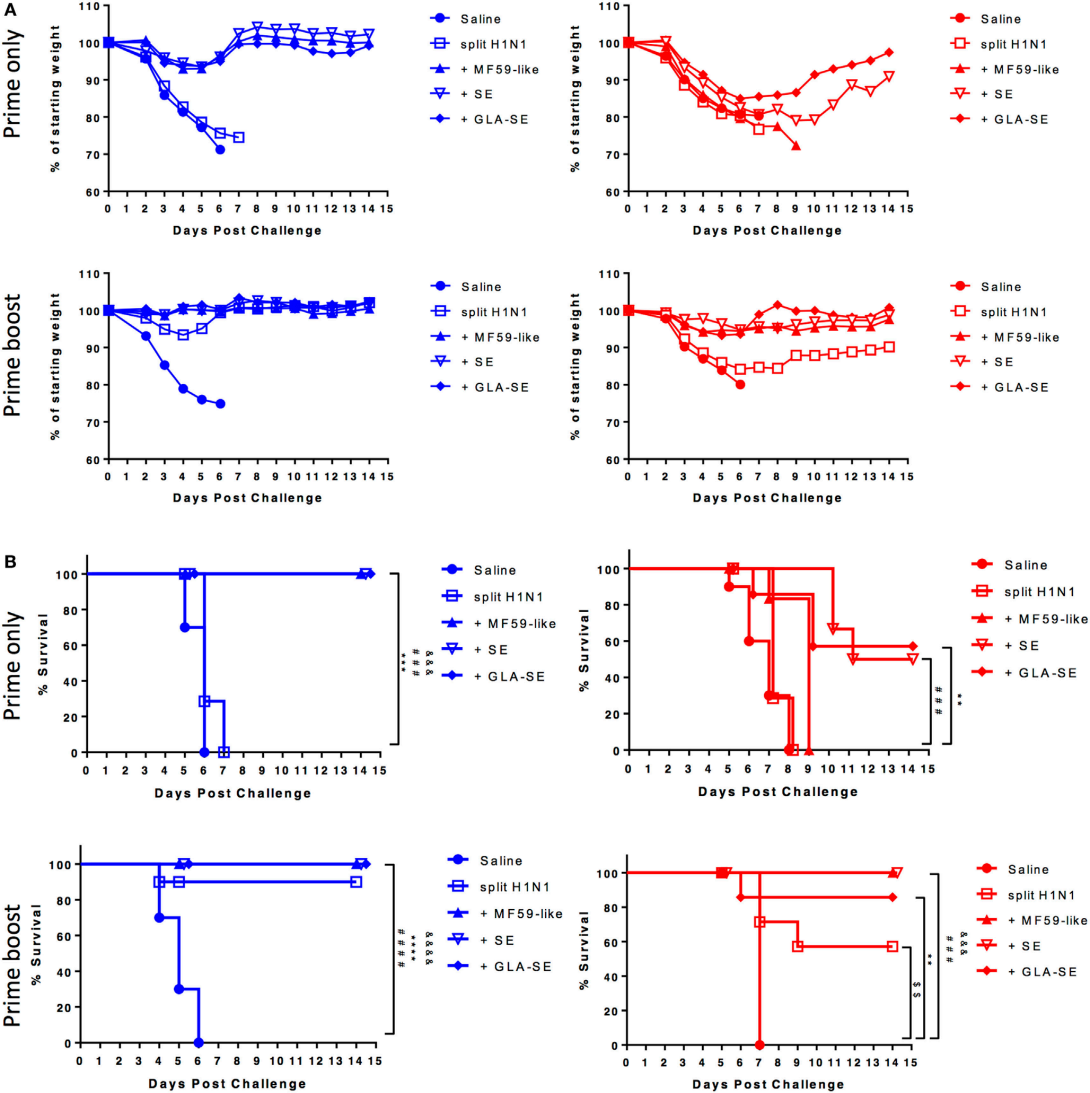
Enhanced body weight and survival in young and aged CB6F1 mice with adjuvanted sH1N1 vaccines after boost immunization. Following immunization with adjuvanted and unadjuvanted sH1N1 vaccines (as shown in Figure 1A) and challenge with 100LD_50_ A/H1N1/California/4/2009, **(A)** the body weight and **(B)** survival were monitored every day over the course of 14 days. Young mice were color-coded as blue; aged mice were color-coded as red. *p-*Values are denoted as follows: ** indicates < 0.01; *** indicates < 0.001; **** indicates < 0.0001. ^$^ compares *p* value between saline vs. sH1N1 groups; ^&^ compares *p* value between saline vs. MF59-like groups; ^#^ compares *p* value between saline vs. SE groups; * compares *p* value between saline vs. GLA-SE groups.

CB6F1 mice were immunized once or twice with 0.01 µg of sH1N1 vaccine either alone or with MF59-like, SE, or GLA-SE adjuvants and then challenged with 100LD_50_ A/H1N1/California/4/2009 (Figure 1A). All mice that received a single immunization lost weight after viral challenge, yet, we found that young mice immunized with MF59-like, SE, and GLA-SE adjuvants could gain weight back by day 5 postinfection (Figure 2A, top left). Conversely, weight loss continued among aged CB6F1 mice until day 7, and only aged mice that received GLA-SE and SE adjuvants gained back weight over time (Figure 2A, top right). Both young and aged mice benefited from two immunizations with adjuvanted sH1N1 vaccines. Young mice given a boost with the adjuvanted vaccines quickly controlled the weight loss at day 3, and aged mice given adjuvanted vaccine only lost approximately 5% of starting weight compared to control animals (Figure 2A, bottom row). Aged mice given saline or sH1N1 vaccine alone lost approximately 20% of starting weight at day 6 (Figure 2A, bottom right). As shown in Figure 2B, survival in saline and sH1N1 young mice, and among all aged animals were severely compromised with one immunization. We observed that most of the young mice (with the exception of the saline group) survived after receiving two immunizations (Figure 2B, bottom left). Survival among aged mice given sH1N1 combined with MF59-like, SE and GLA-SE adjuvants was significantly improved with two immunizations (Figure 2B, bottom right). Our data suggest that increasing the number of immunizations in aged mice from one to two immunizations could considerably improve clinical outcomes following the early stages of influenza infection. Similar to responses in young and aged mice immunized with adjuvanted sH1N1 vaccines, enhanced survival was observed in both young and aged C57BL/6 mice given two immunizations with adjuvanted rH1 vaccines (Figure S1D in Supplementary Material). This is particularly interesting based on the low HAI titers observed in aged mice following a boost immunization. The enhanced clinical outcome in aged mice with adjuvanted rH1 vaccine in the absence of HAI titers suggest that a compensatory immune response is contributing to the protective responses seen in aged mice.

### The GLA-SE-Based sH1N1 Vaccine Promotes the Generation of Cytokine-Producing T Helper Cells, Long-Lived Bone Marrow Plasma Cells (BMPCs) and GC B Cells in Young Mice

**Figure 3:**
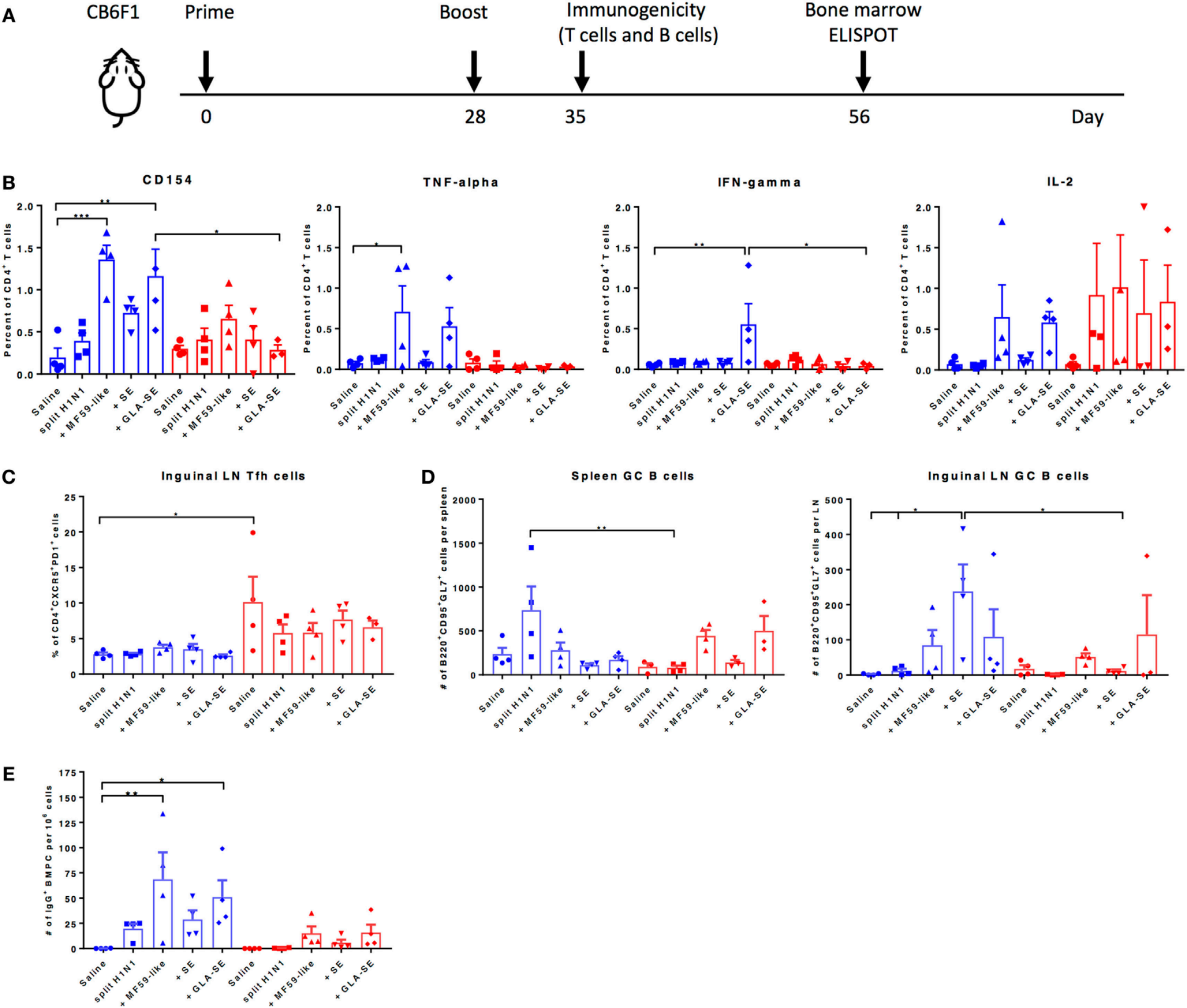
MF59-like and GLA-SE-based sH1N1 vaccine promotes the generation of cytokine-producing T helper cells, GC B cells, and long-lived bone marrow plasma cells in young CB6F1 mice. **(A)** Scheme of slightly modified immunization procedure: CB6F1 mice were immunized i.m. twice, 4 weeks apart and **(B)** the immunogenicity of splenic cytokine-producing CD4^+^ T cells, **(C)** the percentage of CD4^+^CXCR5^+^PD-1^+^ Tfh cells in inguinal LNs, and **(D)** the number of B220^+^CD95^+^GL7^+^ GC B cells in spleen and in inguinal LNs were determined 1 week after the boost immunization with adjuvanted or unadjuvanted sH1N1 vaccines as indicated **(E)** The number of H1-specific BMPCs were determined 4 weeks after a boost immunization. Data from young mice were color-coded as blue; data from aged mice were color-coded as red. * indicates *p* value < 0.05; ** indicates *p* value < 0.01. Error bars indicate mean ± SEM. GC, germinal center; LN, lymph node; Tfh, follicular T helper cells; BMPC, bone marrow plasma cells. Statistical analysis was performed using one-way ANOVA and Tukey’s multiple comparison test except 3E, which used one-way ANOVA and Bonferroni’s test.

We next examined the cellular components that may contribute to protective immune responses in young and aged CB6F1 mice. Young and aged mice were immunized twice with adjuvanted sH1N1 vaccines using a slightly modified strategy, denoted in Figure 3A. The immunogenicity of T cell recall responses and GC B cell responses were determined 7 days following a boost immunization. Splenocytes from young and aged mice with two immunizations were stimulated with rH1 protein, and cytokine production from antigen-specific CD4^+^ T cells were determined. We found that young mice that received both MF59-like or GLA-SE adjuvanted vaccines generated antigen-specific CD4^+^ T cells that produce TNF-α, IL-2, and express CD154. However, only young mice given GLA-SE-adjuvanted vaccine induced IFN-γ-producing CD4^+^ T cells. From aged mice, we detected a minimal percentage of antigen-specific CD4^+^ T cells with increased non-specific CD154 levels (representing activated T cells); however, some mice were able to produce IL-2 (Figure 3B). None of the aged mice induced TNF-α or IFN-γ-producing antigen-specific CD4^+^ T cells following immunization. These data are consistent with our observation in Figure 1A, where aged mice failed to generate Th1-biased IgG2 antibody responses. Also, similar to previous observations (23), we found that aged mice have an increased percentage of non-specific follicular helper T cells (Tfh, CD4^+^CXCR5^+^PD-1^+^) as compared to young mice (Figure 3C). It has been shown that the quality and quantity of neutralizing antibodies is crucial for protective immune responses against viral infection (24). Thus, we examined the induction of GC B cells from spleen and inguinal LNs and long-lived BMPCs in both young and aged mice (Figures 3D,E). Interestingly, as shown in Figure 3D, within the spleen, young mice receiving two immunizations with sH1N1 alone had the highest number of GC B cells, whereas aged mice receiving sH1N1 combined with MF59-like adjuvant and GLA-SE had the highest number of GC B cells (although none of these responses reached statistical significance). The number of B220^+^CD95^+^GL7^+^ GC B cells in the inguinal LN from young mice were enhanced after a boost immunization with all of the adjuvanted sH1N1 vaccines tested, although significant responses were observed only with sH1N1 + SE (Figure 3D). In aged mice, the MF59-like adjuvant induced the highest number of GC B cells in the LN (Figure 3D). We also found that young mice that received MF59-like or GLA-SE adjuvanted sH1N1 vaccines generated a significant number of antigen-specific BMPCs (Figure 3E). Aged mice that received MF59-like and GLA-SE adjuvanted vaccines showed a similar trend to that seen in young mice, although the responses were not statistically significant (Figure 3E).

### *In Vitro* Adjuvant Stimulation of BMDCs and Lung Cells from Aged Mice Exhibit Impaired Cytokine-Producing Ability Compared to Responses from Young Mice

**Figure 4:**
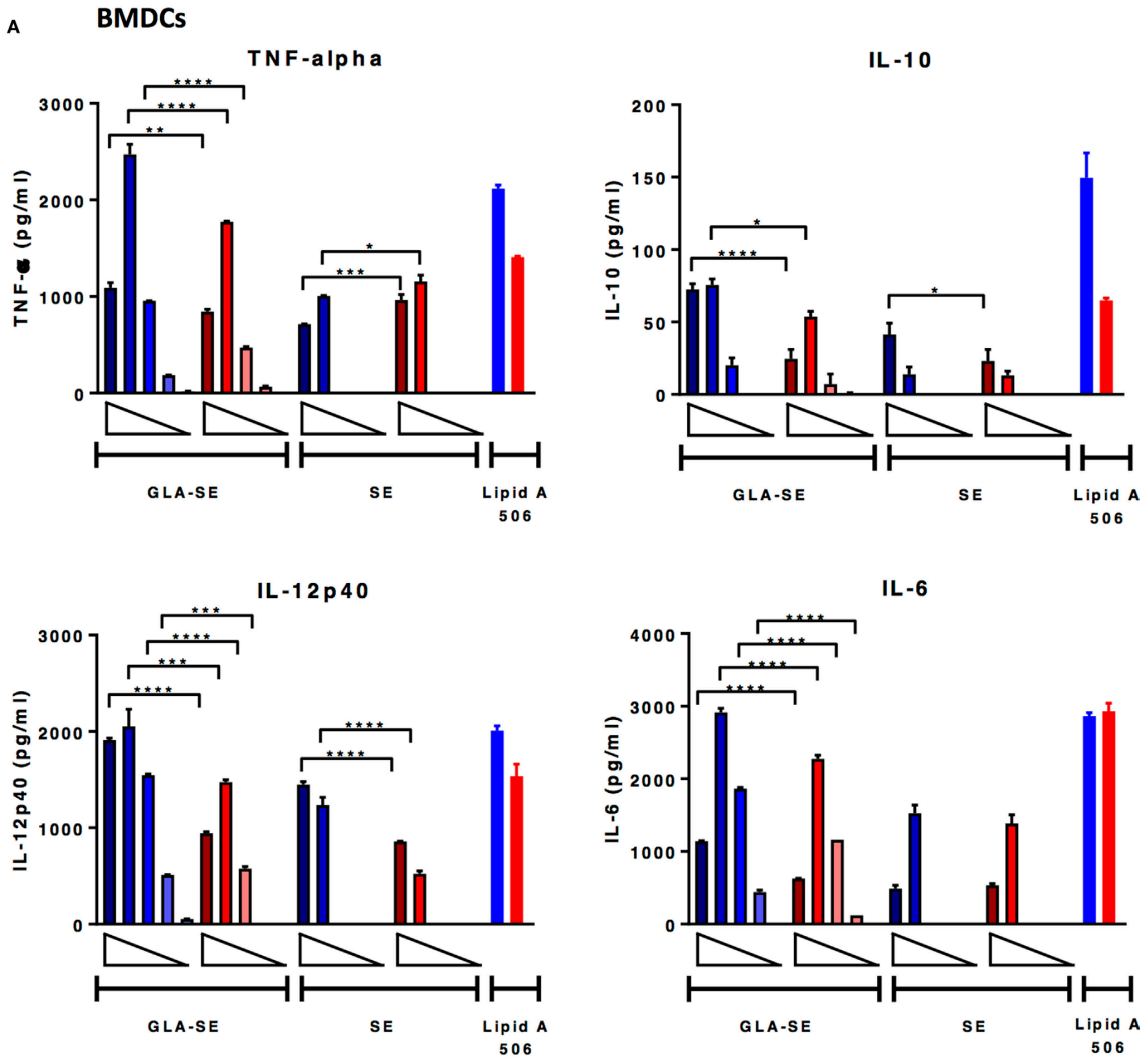

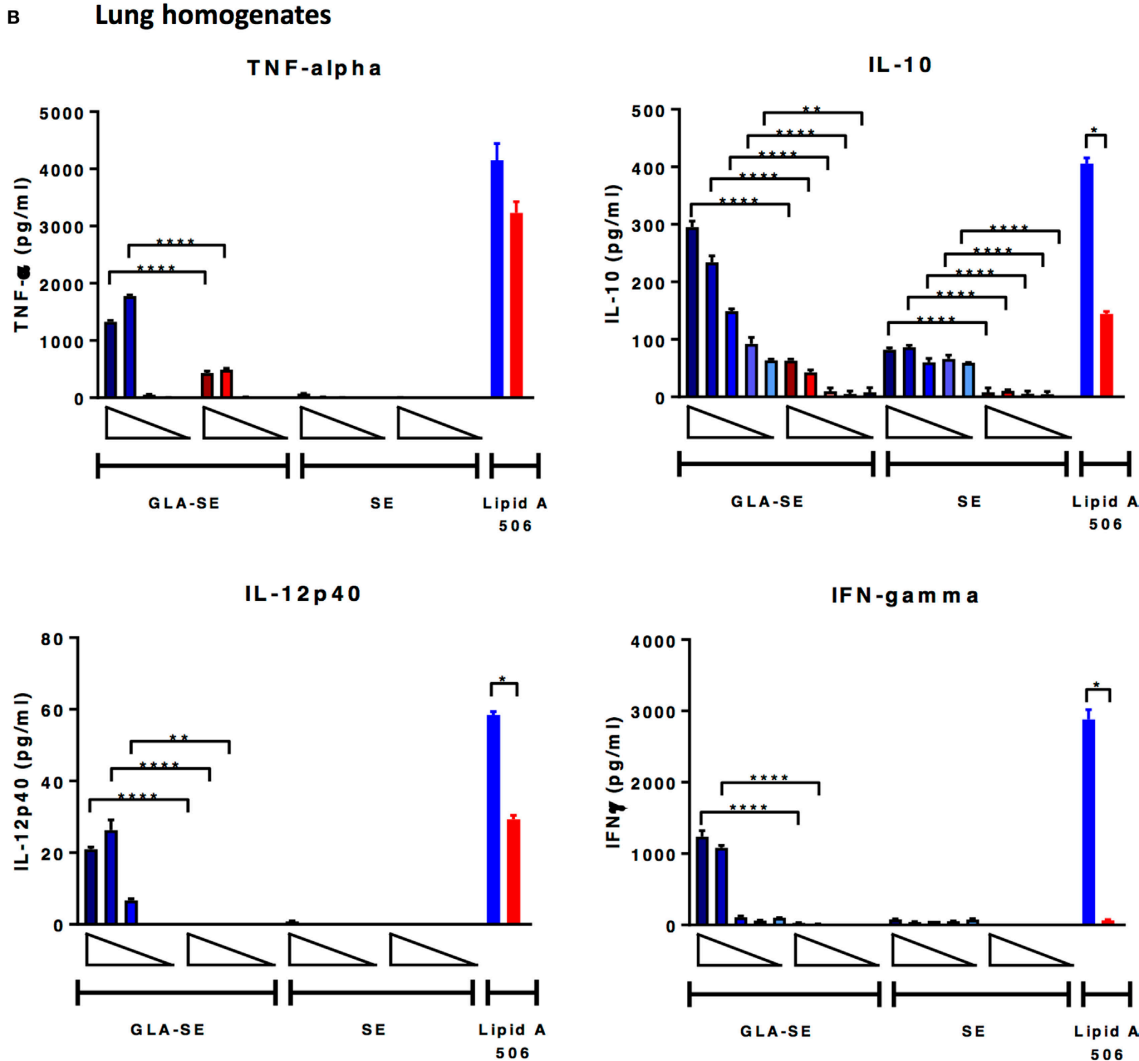
BMDCs from aged mice produce lower levels of cytokine upon GLA-SE stimulation and lung homogenates from aged mice have severely impaired cytokine-producing ability. **(A)** BMDCs and **(B)** lung homogenates from young and aged CB6F1 mice were stimulated with 10-fold dilutions of GLA-SE (2, 20, 200, 2, and 20 µg/ml) or SE (0.00008, 0.0008, 0.008, 0.08, and 0.8%) overnight, followed by harvesting the supernatants the next day for cytokine analysis. Open triangles indicate the concentration from highest (left) to the lowest (right). 10 ng/ml lipid A 506 was used as positive control. Cells from young mice were color-coded as blue; cells from aged mice were color-coded as red. Results are represented as the mean ± SEM. *p-*values are denoted as follows: * indicates < 0.05; ** indicates < 0.01; *** indicates < 0.001; **** indicates < 0.0001. BMDC, bone marrow dendritic cells.

Studies have shown that immune responses are reduced in aged animals due to decreased numbers of APCs and stromal cells (25), increased threshold required for TLR signaling (26), reduced B cell repertoire and humoral responses (7), and a phenomenon where immune organs eventually fill with fat or connective tissues (27). Following our observation that the induction of immune responses to sH1N1 vaccines were impaired in aged mice as compared to young mice, we next tested how adjuvants may affect early innate immune responses from both young and aged mice, characterized by *in vitro* cytokine production from BMDCs and lung homogenates. BMDCs, phenotypically and functionally similar to conventional DCs (28), are the surrogate to induction of systemic adaptive immune responses, whereas stimulated lung homogenates illustrate local immune responses. We tested BMDCs and lung homogenates from both young and aged CB6F1 mice in response to various doses of SE and GLA-SE and found that although BMDCs from aged mice are capable of producing innate inflammatory cytokines upon GLA-SE stimulation, aged cells produced significantly less cytokines compared to those from young animals (Figure 4A). Strikingly, we could scarcely detect inflammatory cytokines in the cell supernatants from GLA-SE-stimulated aged lung homogenates, especially for IL-12p40 and IFN-γ (Figure 4B). Our data suggest that although immunosenescence in aged mice affects innate immune responses both systemically (BMDCs) and locally (lung homogenates), the specific impairment of local immune responses in aged mice could lead to unfavorable and inefficient pathogen clearance at early stages following infection.

### GLA-SE Adjuvanted sH1N1 Vaccine Decreases Viral Load and Prevents Prolonged Lung Inflammation in Young CB6F1 Mice

**Figure 5:**
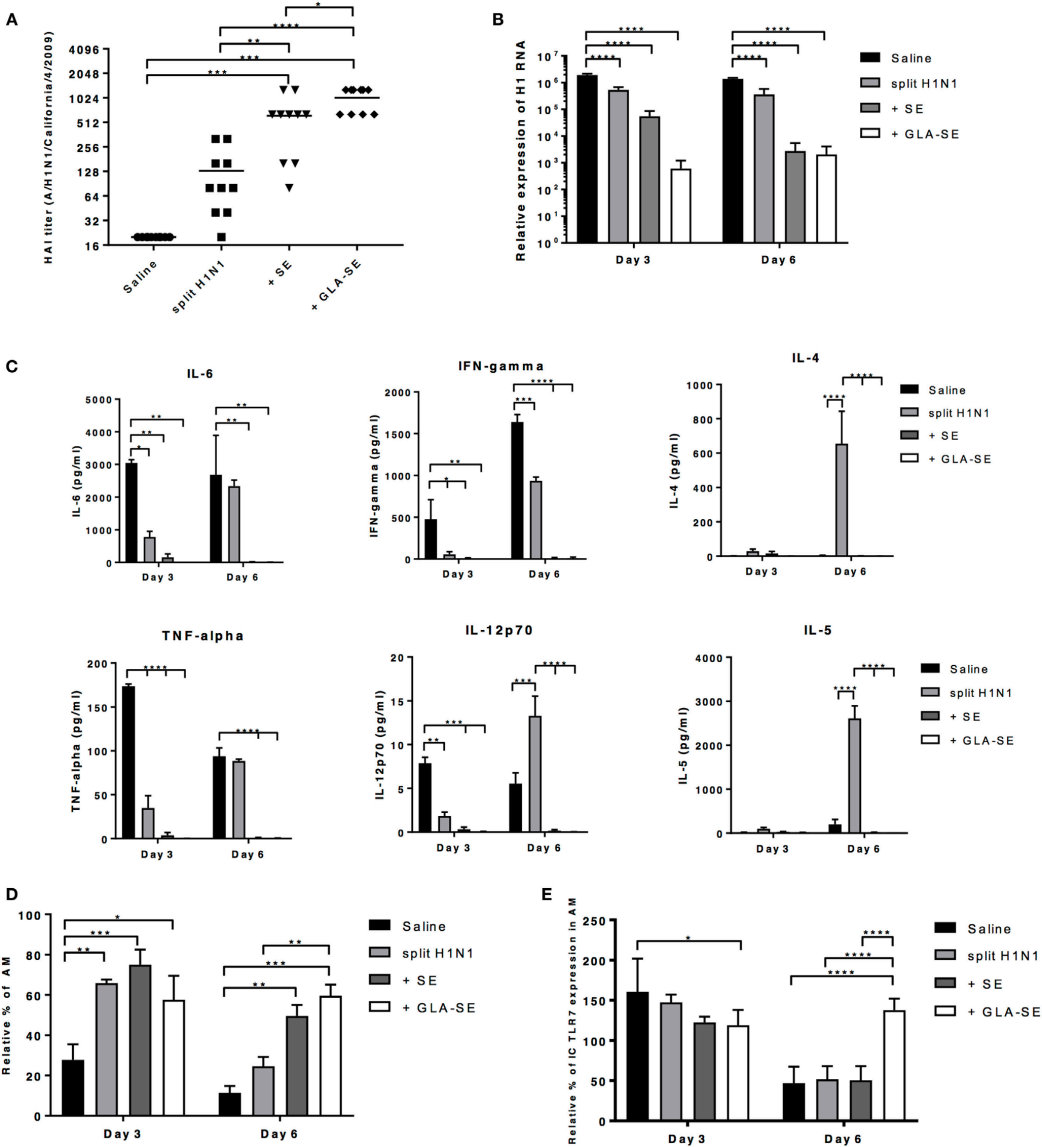
GLA-SE adjuvanted sH1N1 vaccine decreases viral load and prevents prolonged lung inflammation in young CB6F1 mice. Young CB6F1 mice (age of 6–8 weeks old) were immunized once with saline, sH1N1, sH1N1 + SE, or sH1N1 + GLA-SE, and sera were harvested 3 weeks after prime. All mice were challenged with 100LD_50_ A/H1N1/California/4/2009 at day 28 and BALF and lung homogenates were harvested at day 3 and day 6 post-infection. **(A)** Hemagglutination inhibition (HAI) titer against A/H1N1/California/4/2009 was determined. **(B)** The relative expression of H1 was determined by real time Q-PCR. The expression of H1 of each sample was normalized to GAPDH (graph on log10 scale). **(C)** BALF from all infected mice were harvested at day 3 and day 6 postinfection, and the levels of IL-6, IFN-γ TNF-α, IL-12p70, IL-4, and IL-5 cytokines were determined by Luminex. **(D)** The percentage of AMs was determined by flow cytometry and were normalized to uninfected, naïve mice (as 100%). AMs in the lung homogenates were defined as CD11b^−^CD11c^+^Siglec-F^+^NK1.1^−^ population. **(E)** The intracellular expression of TLR7 by AMs was determined by flow cytometry and was normalized to uninfected, naïve mice (as 100%). *p* Values are denoted as follows: * indicates <0.05; ** indicates <0.01; *** indicates <0.001; **** indicates <0.0001. Results are represented as the mean ± SEM. BALF, bronchoalveolar lavage fluid; AM, alveolar macrophages.

Since we observed significant weight change and decrease in survival by day 5 after viral challenge in young and aged mice given either saline or sH1N1 vaccine alone (Figure 2), we hypothesized that mice immunized with adjuvanted vaccines would be able to control local inflammation in the lung during the early infection phase, preventing subsequent mortality. In order to address this hypothesis, young CB6F1 mice were immunized once with saline, sH1N1, sH1N1 + SE, or sH1N1 + GLA-SE were then challenged with 100LD_50_ A/H1N1/California/4/2009. Following challenge, viral loads were determined at days 3 or 6 post challenge. Young CB6F1 mice that received GLA-SE adjuvant generated the highest functional neutralizing antibody titers following one immunization as compared to other groups (Figure 5A). Consistent with our previous data (Figure 1C), mice that received saline or a prime immunization with the sH1N1 vaccine did not generate significant neutralizing antibodies (Figure 5A); in addition, we found that the viral loads were 1,000-fold higher in saline and sH1N1 groups compared to responses in the sH1N1 + GLA-SE group at days 3 and 6 following influenza infection (Figure 5B). Interestingly, the sH1N1 + SE group had 100-fold higher viral load than the sH1N1 + GLA-SE group at day 3, but no differences in virus titer were observed at day 6, suggesting that sH1N1 adjuvanted with GLA-SE is superior to sH1N1 adjuvanted with SE, and animals immunized with sH1N1 + GLA-SE were able to clear viral infection earlier after influenza challenge (Figure 5B).

Next, we examined the inflammatory cytokines and chemokines in bronchoalveolar lavage fluid (BALF) from young mice at day 3 or 6 after viral challenge. To our surprise, we could not detect any inflammatory cytokines in BALF from mice that received either SE or GLA-SE adjuvants (Figure 5C). IL-6, TNF-α, IFN-γ, and IL-12p40 were detected in the BALF from saline and sH1N1 groups at day 3 and 6, indicating that a continuous inflammatory response occurs in the lungs of these mice. Notably, Th2-biased cytokine IL-4, IL-5, and chemokine Eotaxin (data not shown) in the BALF were detected at day 6 from mice that received the sH1N1 vaccine alone. These data corroborate our previous reports that immunization with protein only, or without proper Th1-driving adjuvants, leads to Th2-biased immune responses (29). It has also been reported that alveolar macrophages (AMs) are crucial for lung homeostasis and are the first line cells capable of detecting lung pathogens including bacteria, viruses, in addition to allergens and foreign particles (30). Thus, we examined whether AMs from young mice given a prime immunization were affected after exposure to A/H1N1/California/4/2009 challenge. Interestingly, we found that unvaccinated, young CB6F1 mice (saline group) had dramatically decreased percentages of AMs at day 3 of infection as compared to the other groups. At day 6, both saline and sH1N1 groups had a significantly decreased percentage of AMs as compared to adjuvanted groups, suggesting that AMs are potentially being eliminated by the viral infection (31) (Figure 5D). Similarly, a reduction in the percentage of AMs following influenza infection correlate with an increased viral load within the lung. As shown in Figure S2 in Supplementary Material, we found that in young mice given one immunization, only mice that received the sH1N1 + GLA-SE vaccine maintain AM homeostasis and prevent inflammatory cell infiltration (including eosinophils, neutrophils, inflammatory Mϕs, and NK cells) in the lung at both day 3 and 6 post-infection. After influenza infection, cells such as AMs that express TLR7 are able to detect single-strand RNA fragments released from viral particles. We found that expression of TLR7 on AMs were relatively consistent among all groups at day 3; however, the TLR7 levels were significantly decreased in saline, sH1N1 and sH1N1 + SE groups by day 6 as compared to sH1N1 + GLA-SE group (Figure 5E). Overall, our data suggest that the GLA-SE adjuvanted sH1N1 vaccine provides superior protection in young mice by clearing influenza virus early after infection, and through maintenance of AMs and TLR7 expression levels in the early infection phase.

## Discussion

In this study, the influence of different adjuvants on the efficacy of two types of seasonal influenza vaccine: a rH1 protein vaccine, and a sH1N1 vaccine was evaluated. Adjuvants examined include two squalene-based oil-in-water emulsions [a MF59-like adjuvant and SE (a stable emulsion adjuvant)] and a synthetic TLR4 agonist formulated with SE (GLA-SE). In CB6F1 mice, we showed that both the MF59-like adjuvant and GLA-SE plus sH1N1 vaccine contribute to Th1-biased vaccine-specific IgG2 immune responses following a boost immunization, whereas significantly higher IgG2c titers were not induced in aged mice. HAI titers were, however, induced in both young and aged CB6F1 mice after two immunizations. In contrast, in C57BL/6 mice, we observed an increase in vaccine-specific IgG1 and IgG2c responses following a boost immunization with all of the adjuvanted rH1 vaccines tested. However, whereas HAI titers were above the 1:40 threshold with adjuvanted rH1 vaccines in young C57BL/6 mice [HAI titers above a titer of ≥1:40 in humans is associated with a 50% reduction in seasonal influenza infection and is considered a correlate of protection (32, 33)], HAI titers were not increased in aged mice. Following a boost immunization, aged C57BL/6 and CB6F1 mice induced significant levels of either vaccine-specific IgG2c or HAI titers, respectively, leading to protection against influenza challenge. This observation is of interest as cellular immunity is known to decline with age and compensatory mechanisms induced by adjuvants may aid protection in the elderly. Recently, the induction of IgG2 antibodies have been a focus of interest for protection against infection, including influenza infection. An M2e-specific IgG2c monoclonal antibody was reported to protect mice against influenza infection more effectively than an IgG1 monoclonal due to Fc activation properties (34).

The use of adjuvants as a strategy for enhancing the quality and magnitude of protective antibody responses to influenza vaccines is not a new concept. We have shown previously that the addition of adjuvants such as GLA-SE are able to increase dose-sparing properties and broaden HAI titers against drifted influenza viruses (12). In this manner, applying the GLA-SE adjuvant to influenza vaccine design addresses a noted and significant complication of reduced vaccine-specific antibody responses observed in aged individuals (9). GLA-SE combined with a recombinant H5N1 avian influenza vaccine (rH5) also increases the breadth of the antibody responses and provides protection against a heterosubtypic influenza virus, compared to the vaccine combined with SE (14).

One of the benefits of using GLA-SE as a vaccine adjuvant is the Th1-inducing innate responses that promote protective adaptive Th1-mediated cellular and humoral immunity (12, 29, 35). Characterization of this pure synthetic TLR4 agonist adjuvant in comparison to another TLR4 agonist, monophosphoryl lipid A (MPL), which is derived naturally from the bacterium *Salmonella minnesota*, has been shown following stimulation of DCs from both humans and mice (36). Both agonists were shown to increase DC activation and maturation and induce proinflammatory cytokines and chemokines (12). In elderly humans, the responses of monocyte-derived DCs stimulated with GLA-SE appear intact (16). Here, we add to this paradigm and demonstrate that BMDCs derived from aged mice respond to GLA-SE stimulation; however, cytokine responses are lower than observed following GLA-SE stimulation of young BMDCs.

Peripheral blood mononuclear cells from elderly patients stimulated with GLA-SE and a split virus vaccine have also been shown to increase the ratio of IFN-γ:IL-10, in addition to granzyme B, further supporting this as a potentially effective candidate adjuvant for use in the elderly (19). In our study, MF59-like adjuvant was also capable of promoting vaccine-specific CD4^+^ T cell responses, GC B cells, and induction of long-lived BMPCs, and like the SE adjuvant, was able to induce HAI titers after a boost immunization leading to protection in mice against challenge with H1N1. The history, safety, and efficacy of the MF59 adjuvant has been recently reviewed (17, 18).

One of the most striking observations in our study was the differential cytokine responses to adjuvant in aged vs. young lung homogenates. The *in vitro* stimulatory response of GLA-SE on lung homogenates from young mice led to the induction of TNF-α, IL-12p40, IFN-γ, and IL-10, whereas only TNF-α and IL-10 were induced in samples from aged mice. This potential local impairment (within the lung) could have substantial consequences following acute infection with influenza virus and other pulmonary infections.

In order to determine the protective mechanism of action of sH1N1 adjuvanted with GLA-SE within the lungs of young mice, we further characterized the pulmonary responses following H1N1 infection, including assessment of viral load and inflammatory responses. We observed less virus in the lung at day 3 post challenge, in mice immunized once with sH1N1 combined with GLA-SE, and decreased levels of virus at day 6 post challenge in mice immunized with SE compared to saline or sH1N1 vaccine only. The magnitude of the neutralizing HAI titer was also highest in mice given the sH1N1 + GLA-SE. Furthermore, a drastic reduction in the inflammatory responses within the lungs of mice immunized with the sH1N1 + GLA-SE was observed compared to the Th2-biased inflammatory responses that were seen in the sH1N1 vaccine alone group. At day 6 of infection, the percentage of AMs was also significantly higher in the sH1N1 + GLA-SE group compared to both saline and sH1N1 immunized mice. Interestingly, TLR7 expression within AMs was also significantly higher in the sH1N1 + GLA-SE-treated mice compared to mice that were immunized with sH1N1 vaccine only and sH1N1 + SE. Recently, Wong et al. reported that AMs from aged mice have an intrinsically impaired function to limit lung damage during influenza viral lung infection and suggested that enhancing the function of AMs may improve outcomes in elderly individuals infected with respiratory viruses (31). It will be of great interest to determine if administration of Th1-inducing vaccines in aged animals can preserve the numbers and functions of AMs during acute phases of infection, and how this might reduce the morbidity and mortality in aged individuals. This significant hypothesis warrants further exploration. We have previously shown, in both mice and non-human primates, that Fluzone^®^ (a trivalent inactivated influenza vaccine) adjuvanted with GLA-SE, increases the magnitude of HAI titers, induces Th1 cellular immune responses, and enhances cross-reactive antibody responses to drifted influenza strains (12). Our current work further addresses additional attributes of GLA-SE-adjuvanted influenza vaccines in the context of increased age.

Finally, the CDC estimates that during the 2015–2016 influenza season, the elderly (making up only 15% of the overall US population) accounted for half or more of hospitalizations associated with influenza and 64% of deaths associated with pneumonia and influenza (37). Our data suggest that the staggering burden of morbidity and mortality due to influenza infection in the elderly population may be alleviated by further detailed and mechanistic selection of proper adjuvants to be included in seasonal vaccines.

## Ethics Statement

These studies were carried out in accordance with the regulations and guidelines of the IDRI animal care and use committee (IACUC). The animal protocol was approved by the IACUC committee.

## Author Contributions

SB and FCH wrote and prepared the manuscript; SB, FCH, EG, SL, and RC edited the manuscript; SB, NH, EG, RC, and FCH designed the experiments; NH, EG, and BG did HAI and viral infection; FCH and JG did Luminex assays; FCH did the quantitative PCR and flow analysis; EL and LH did *in vitro* stimulation of BMDCs and lung homogenates; and SB, FCH, and SL did the data analyses.

## Conflict of Interest Statement

SR is a founder of, and holds an equity interest in, Immune Design Corporation, a licensee of certain rights associated with GLA. All other authors have no financial conflicts of interest.
