# Supplementary material for: Improved Immune Responses in Young and Aged Mice with Adjuvanted Vaccines against H1N1 Influenza Infection
Source: Front Immunol. 2018 Feb 19;9:295. doi: 10.3389/fimmu.2018.00295 (PMC5826078; doi:10.3389/fimmu.2018.00295)
Supplement: Supplementary file 1 [file image_1.PDF]

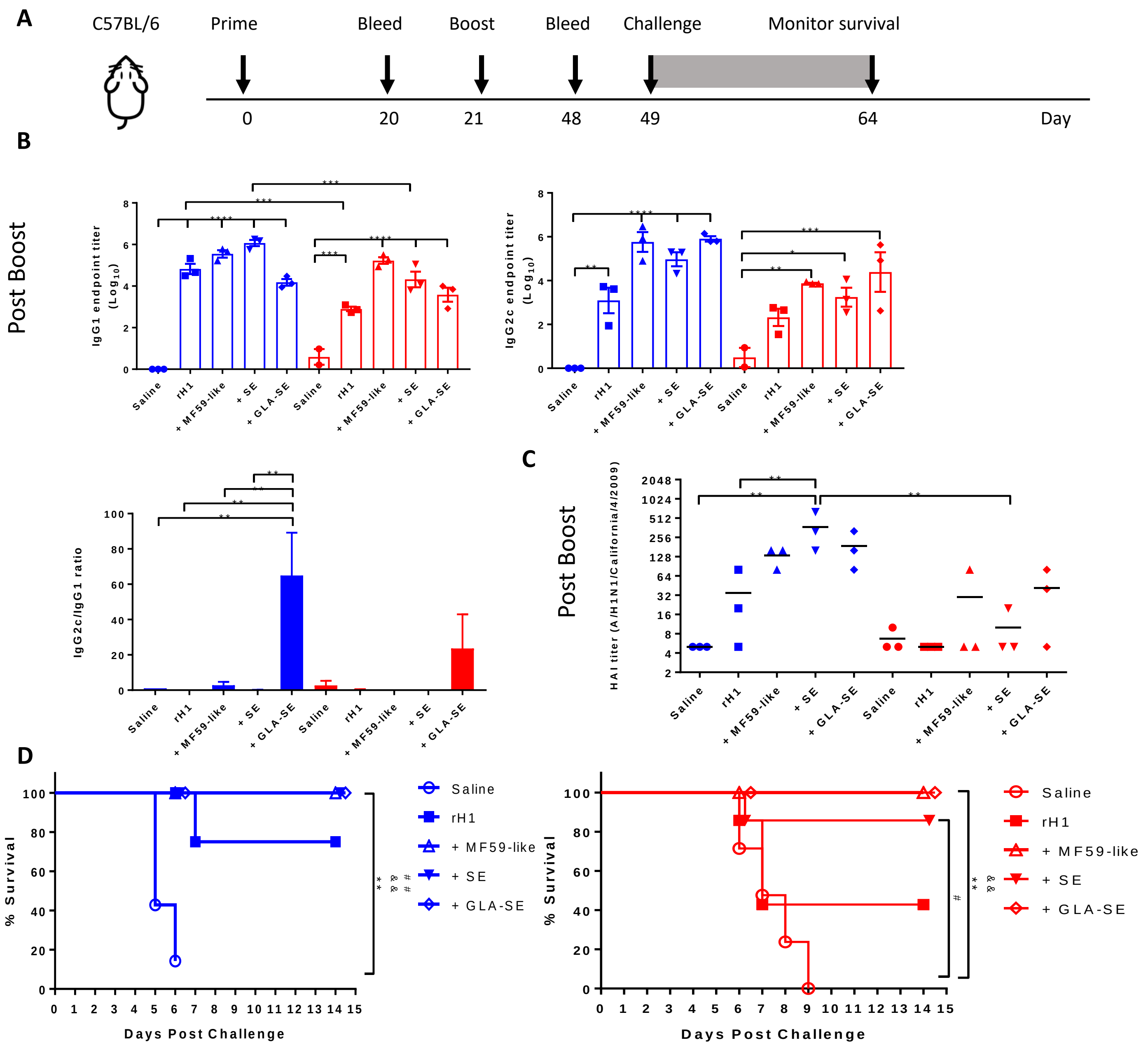


**Supplemental Figure 1. Adjuvanted rH1 vaccines enhance IgG2c:IgG1 ratios and protection in young and aged C57BL/6 mice following a boost**

**(A)** Scheme of immunization procedure: C57BL/6 mice were immunized i.m. twice, three weeks apart, and antibody analysis was determined on sera collected four weeks following the boost. All mice were challenged with $100LD_{50}$ A/H1N1/California/4/2009 at day 49 and their physical condition was monitored over 14 days. Young mice (blue) and aged mice (red) are shown. **(B)** Sera collected from saline, rH1, rH1+MF59-like, rH1+SE and rH1+GLA-SE groups were analyzed for H1-specific IgG1 and IgG2c endpoint titers. Results are represented as the mean endpoint titer ($\log_{10}$) ± SEM. *p* values are denoted as follows: * indicates <0.05; ** indicates <0.01; *** indicates < 0.001; **** indicates < 0.0001. **(C)** Sera harvested from mice after a boost (day 48) immunization were analyzed for HAI titers. An HAI titer of 5 represents responses below the assay detection limit. ** indicates *p* value <0.01. **(D)** Survival was monitored over the course of 14 days among all groups of young mice (left panel, blue) and aged mice (right panel, red). $^{\&\&}$ indicates *p* value <0.01 between saline vs. MF59-like groups; $^{\#\#}$ indicates *p* value <0.01 between saline vs. SE groups; ** indicates *p* value <0.01 between saline vs. GLA-SE groups.
