# Supplementary material for: Improved Immune Responses in Young and Aged Mice with Adjuvanted Vaccines against H1N1 Influenza Infection
Source: Front Immunol. 2018 Feb 19;9:295. doi: 10.3389/fimmu.2018.00295 (PMC5826078; doi:10.3389/fimmu.2018.00295)
Supplement: Supplementary file 2 [file image_2.PDF]

A

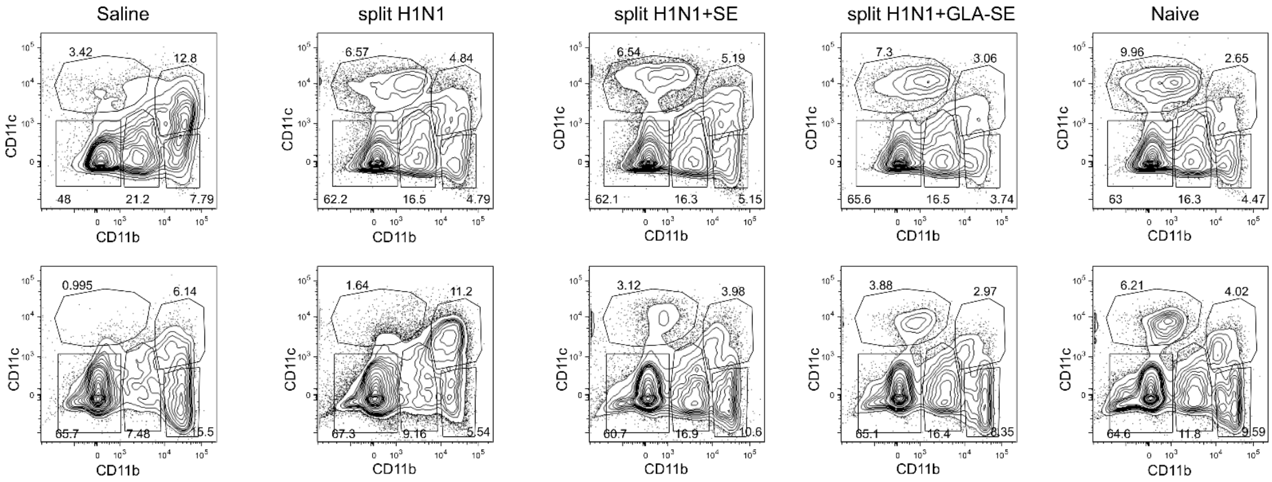

B

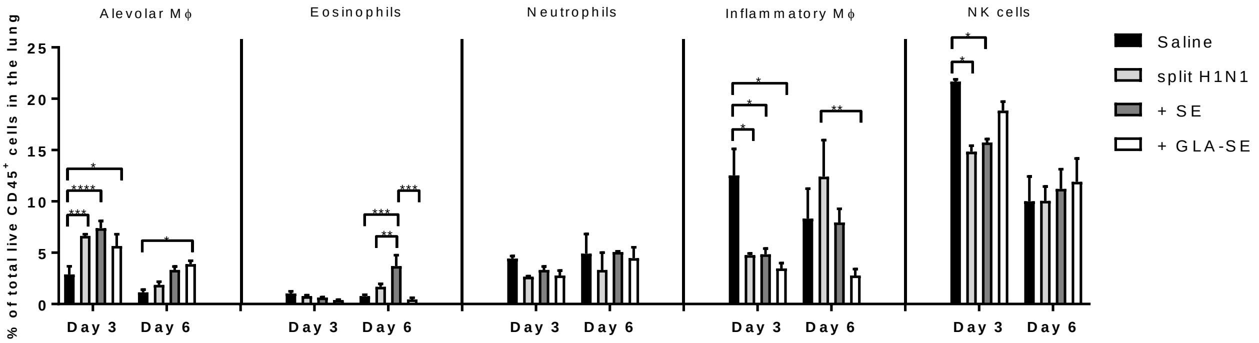

Supplemental Figure 2. Split H1N1 vaccine adjuvanted with GLA-SE maintains AM homeostasis and prevents inflammatory cell infiltration in the lung during the early infection phase

(A) Phenotypic analysis of cell populations in the lungs from young CB6F1 mice. Representative flow plots of cell populations in the lungs of naïve mice (no infection), control mice (saline), or immunized mice at day 3 and day 6 post H1N1 infection. Mice were immunized once with sH1N1, sH1N1+SE, sH1N1+GLA-SE, or were injected with saline, and were challenged 3 weeks later with 100LD$_{50}$ A/H1N1/California/4/2009. Cells are pre-gated on singlet, live and CD45^{+} populations. Alveolar macrophages (Mϕ) are defined as CD11b$^{lo/-}$CD11c^{+}Siglec-F^{+} cells; inflammatory Mϕ are defined as CD11b^{hi}CD11c^{+}Siglec-F^{-} cells; granulocytes including eosinophils and neutrophils are defined as CD11b^{hi}CD11c^{-}Siglec-F^{+}Ly6G^{mid} and CD11b^{hi}CD11c^{-}Siglec-F^{-}Ly6G^{+}, respectively; NK cells are defined as CD11b^{mid}CD11c^{-}NK1.1^{+}. **(B)** Cellular percentages of alveolar Mϕ, eosinophils, neutrophils, inflammatory Mϕ and NK cells in total live CD45^{+} cells in the lung. Results are represented as the mean ± SEM (n=4 per group). *p* values are denoted as follows: * indicates <0.05; ** indicates <0.01; *** indicates < 0.001; **** indicates < 0.0001.
